# Supplementary material for: Hierarchical Flows of Human Cortical Activity
Source: bioRxiv. 2026 Mar 19:2026.03.19.712872. Preprint. [Version 1] doi: 10.64898/2026.03.19.712872 (PMC13015493; doi:10.64898/2026.03.19.712872)
Supplement: 1 [file NIHPP2026.03.19.712872V1-supplement-1.pdf]

bioRxiv preprint doi: https://doi.org/10.64898/2026.03.19.712872; this version posted March 19, 2026. The copyright holder for this preprint (which was not certified by peer review) is the author/funder, who has granted bioRxiv a license to display the preprint in perpetuity. It is made available under aCC-BY 4.0 International license.

Supplementary Material

Validation datasets: simulations, artifact controls, and replication

To validate the cortical-flow framework, we combined synthetic simulations with known ground-truth propagation, targeted artifact-control analyses, and replication of the core findings in an independent dataset (OMEGA).

Methodological validation: simulation of cortical propagation patterns

To evaluate directional accuracy, we simulated biologically plausible cortical propagation on a curved surface using the vectorial heat equation (Lefèvre et al., 2009). This framework extends the classical scalar heat equation to vector fields defined on manifolds and provides smooth, spatially coherent trajectories across cortical geometry.

We begin with the classical parabolic partial differential equation (PDE) defined on a 2D Euclidean domain Ω:

$$\frac{\partial V}{\partial t} = \Delta \boldsymbol{V}, \quad \boldsymbol{V} = 0 \text{ on } \partial\Omega \tag{1}$$

Where the vectorial Laplacian is defined as:

$$\Delta \boldsymbol{V} = \nabla(div\boldsymbol{V}) - rot(rot\boldsymbol{V}) \tag{2}$$

Rather than computing this expression explicitly on the manifold M, we adopt a variational formulation of the vectorial heat equation, which is more computationally tractable and enables numerical integration over curved surfaces. The evolution of the vector field $\boldsymbol{V}$ is governed by:

$$\frac{\partial}{\partial t}\int_{\mathcal{M}} g(\boldsymbol{V}, W)\, d\mu = -a_2(\boldsymbol{V}, W) \tag{3}$$

where $g(\cdot,\cdot)$ denotes the Riemannian metric, $d\mu$ is the volume form on M, $W \in T\mathcal{M}$ is a test vector field, and $a_2(\boldsymbol{V}, W)$ is a bilinear form involving covariant derivatives of $\boldsymbol{V}$ and W. This vectorial diffusion process promotes smoothness and spatial coherence, properties required for biologically realistic simulations of cortical activity propagation.

Using this framework, we simulated large-scale propagation patterns along three cardinal axes: posterior→anterior, anterior→posterior, and inferior→superior (Figure 1). Ground-truth direction at each vertex was defined by the displacement vector between successive time points. To assess statistical significance, we implemented a hemisphere-constrained spherical rotation ("spin") null model that preserves spatial autocorrelation. Each trajectory was randomly rotated 1,000 times across the cortical

bioRxiv preprint doi: https://doi.org/10.64898/2026.03.19.712872; this version posted March 19, 2026. The copyright holder for this preprint (which was not certified by peer review) is the author/funder, who has granted bioRxiv a license to display the preprint in perpetuity. It is made available under aCC-BY 4.0 International license.

surface, generating a null distribution of directional vectors. Observed mean directions and angular deviations were compared against this null, yielding empirical p_{spin} values.

Cortical-flow estimates recovered the ground-truth directions across all simulated conditions (Supplementary Fig. 3). With 90° denoting posterior-to-anterior and 0° denoting superior-to-inferior directions, the posterior-to-anterior simulation yielded a mean estimated direction of 86.81° (95% CI, 73.64°–99.99°; mean angular error 0.97°), the anterior-to-posterior simulation yielded 275.69° (95% CI, 259.51°–291.85°; mean angular error 3.67°), and the inferior-to-superior simulation yielded 157.56° (95% CI, 142.09°–173.61°; mean angular error 11.81°). All recovered directions differed significantly from the spin-based null (pspin < 0.05).

Artifact control analysis

To evaluate whether transient physiological artifacts influenced cortical-flow direction or kinetic energy, we performed targeted control analyses using epochs locked to eyeblinks and heartbeats in the post-QC OMEGA sample (N = 83). Blink onsets were identified from the vertical EOG, and heartbeat onsets were identified from ECG.

For each blink, we extracted epochs spanning -300 to +300 ms and processed the resulting eye-movement event-related fields with the cortical-flow framework. Global and parcellated flow directions were compared between these epochs and artifact-free data, and local directional structure was assessed with the Hodges-Ajne test using FDR correction across regions.

We repeated the same procedure for cardiac artifacts using epochs spanning -50 to +50 ms around each R-peak.

To assess preprocessing effects directly, cortical-flow metrics were also compared before and after signal-space projection (SSP) within artifact-free resting segments.

Blink epochs exhibited a reproducible biphasic pattern, with anterior-to-posterior propagation at blink onset followed by a posterior-to-anterior rebound at blink offset (Supplementary Movie 8). Heartbeat epochs showed the converse pattern (Supplementary Movie 9). Mean onset angles were 15.1° (95% CI, 13.6°–16.5°) for blinks and 188.7° (95% CI, 186.0°–191.3°) for heartbeats.

After SSP correction, this directional structure was abolished and angular distributions no longer differed from uniformity (pperm > 0.05). Kinetic-energy transients visible around blink and heartbeat events were likewise removed, and post-SSP values no longer differed from artifact-free resting segments (paired t test, p > 0.05).

bioRxiv preprint doi: https://doi.org/10.64898/2026.03.19.712872; this version posted March 19, 2026. The copyright holder for this preprint (which was not certified by peer review) is the author/funder, who has granted bioRxiv a license to display the preprint in perpetuity. It is made available under aCC-BY 4.0 International license.

These analyses indicate that physiological artifacts can induce spurious propagation and kinetic-energy transients, but that SSP effectively removes these effects.

Replication analysis (OMEGA)

To test generalizability, we repeated the core analyses in an independent resting-state MEG sample from the OMEGA repository (N = 83), using identical preprocessing and metric definitions.

Broadband activity (0.6–92.6 Hz) again exhibited sagittal posterior-to-anterior propagation, with a group-mean angle of 74.6° (95% CI: 72.8°–76.4°, $p_{perm} < 0.001$). Flow vectors remained preferentially aligned with the principal functional hierarchy, and angular differences showed a bimodal distribution (Hartigan dip, $p_{spin} < 0.001$), indicating co-existing hierarchy-aligned and hierarchy-opposed streams.

Frequency-specific bias replicated: slow activity (1–13 Hz) showed a higher incidence of hierarchy-aligned propagation than beta activity (13–30 Hz) (paired $t = 2.80$, $p = 0.006$, *Cohen's d* = 0.35).

The spatial pattern of kinetic energy mirrored Cam-CAN, with higher values in posterior sensory cortex decreasing toward anterior association areas, yielding a negative correlation with functional hierarchy rank ($r = -0.66$, $p_{spin} < 0.001$). Kinetic energy also correlated negatively with a myelination index derived from T1w/T2w ratios ($r = -0.66$, $p_{spin} < 0.001$).

Stable-state dwell times increased along the same posterior-to-anterior hierarchy ($r = 0.30$, $p_{spin} < 0.001$) and correlated positively with intrinsic neuronal timescales extracted from spectral knees ($r = 0.51$, $p_{spin} < 0.001$).

Together, the OMEGA replication confirms that sagittal propagation, hierarchy alignment, frequency-specific directional biases, and frequency-resolved kinetic gradients generalize across datasets and acquisition platforms.

bioRxiv preprint doi: https://doi.org/10.64898/2026.03.19.712872; this version posted March 19, 2026. The copyright holder for this preprint (which was not certified by peer review) is the author/funder, who has granted bioRxiv a license to display the preprint in perpetuity. It is made available under aCC-BY 4.0 International license.

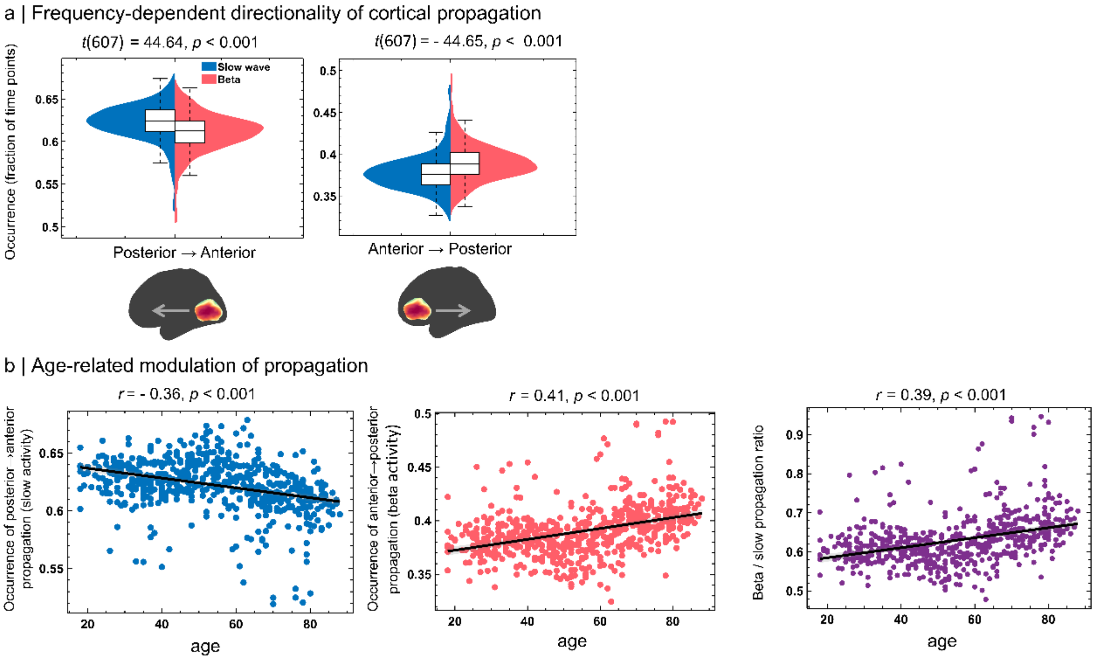

Supplementary Figure 1: Age-related changes in cortical propagation direction across frequency bands.

a | Conceptual model of frequency-specific propagation patterns: This schematic illustrates the hypothesized dominant propagation trajectories of spontaneous cortical activity across frequency bands. Slow-frequency activity (blue) predominantly propagates from posterior to anterior regions, reflecting bottom-up integration, while β-frequency activity (red) tends to propagate in the opposite direction, consistent with top-down control.

b | Frequency-specific propagation direction distributions: Violin plots show the group-level distributions of propagation direction frequencies across participants, comparing slow and β bands along two sagittal axes:

- Left: Slow activity exhibits significantly more frequent posterior-to-anterior propagation ($t = 44.64$, $p < 0.0001$).
- Right: Β activity shows significantly greater anterior-to-posterior propagation ($t = –44.65$, $p < 0.0001$).

These findings confirm a frequency-dependent reversal in preferred propagation direction.

c | Lifespan changes in propagation direction: Scatter plots display how propagation direction varies with age:

- Left: Posterior-to-anterior propagation of slow activity declines with age ($r = –0.36$, $p < 0.0001$).

bioRxiv preprint doi: https://doi.org/10.64898/2026.03.19.712872; this version posted March 19, 2026. The copyright holder for this preprint (which was not certified by peer review) is the author/funder, who has granted bioRxiv a license to display the preprint in perpetuity. It is made available under aCC-BY 4.0 International license.

- Middle: Anterior-to-posterior propagation of β activity increases with age ($r = 0.41$, $p < 0.0001$).
- Right: The β-to-slow directional ratio increases with age ($r = 0.39$, $p < 0.0001$), indicating a shift toward top-down dynamics in aging.

bioRxiv preprint doi: https://doi.org/10.64898/2026.03.19.712872; this version posted March 19, 2026. The copyright holder for this preprint (which was not certified by peer review) is the author/funder, who has granted bioRxiv a license to display the preprint in perpetuity. It is made available under aCC-BY 4.0 International license.

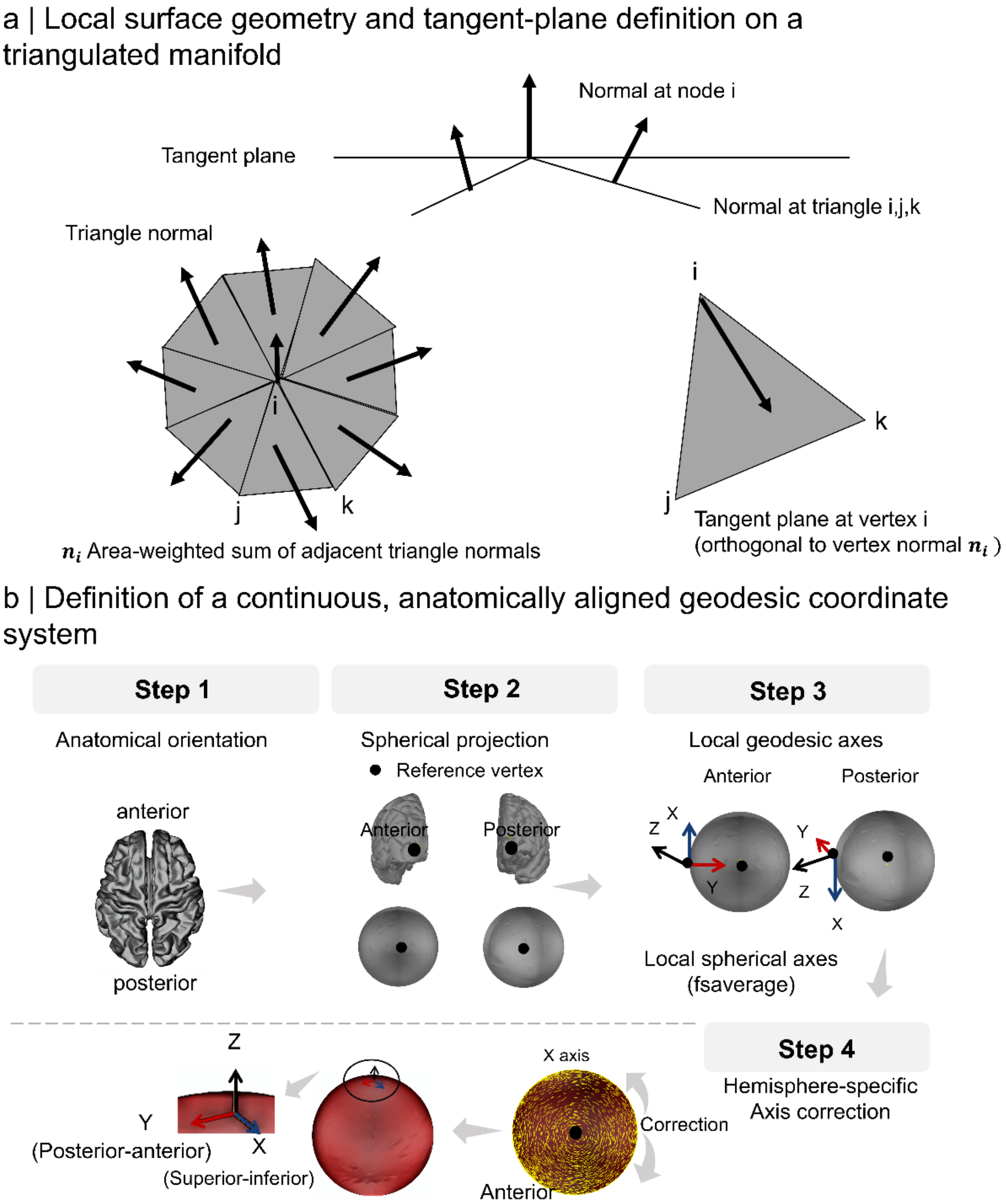

Supplementary Figure 2: Cortical Riemannian manifold and geodesic coordinate system.

a | Riemannian manifold: Schematic illustration of a 2D Riemannian manifold discretized as a triangular cortical mesh in 3D space. At each vertex, a local tangent plane is computed from neighboring faces. The surface normal vector n_p is derived via the cross product of local basis vectors in the plane. This construction enables the application of differential operators, such as gradients and vector field flow, to characterize propagation of neural activity across the surface.

b | **Geodesic coordinate system:** This figure illustrates the procedure used to define a consistent, anatomically grounded reference frame for computing and interpreting

bioRxiv preprint doi: https://doi.org/10.64898/2026.03.19.712872; this version posted March 19, 2026. The copyright holder for this preprint (which was not certified by peer review) is the author/funder, who has granted bioRxiv a license to display the preprint in perpetuity. It is made available under aCC-BY 4.0 International license.

cortical flow vectors across the cortical surface. For each hemisphere, we defined the posterior-anterior axis using the most posterior and most anterior vertices on the *fsaverage* surface. These two points define a posterior-to-anterior Y-axis, which is then projected onto the spherical representation of the cortical manifold. Next, a surface-based X-axis is defined as orthogonal to the Y-axis in the superior–inferior direction, and a Z-axis is derived accordingly to complete the local 3D Cartesian coordinate system (X, Y, Z) at each vertex. To preserve directional consistency across hemispheres—particularly across lateral and medial surfaces—a coordinate flip is applied on one hemisphere to align the anatomical orientations of X across the entire cortex. This anatomically informed, geodesic-based coordinate system enables reliable and uniform interpretation of cortical flow directionality, facilitating group-level comparisons and robust alignment with macroscale cortical hierarchies.

bioRxiv preprint doi: https://doi.org/10.64898/2026.03.19.712872; this version posted March 19, 2026. The copyright holder for this preprint (which was not certified by peer review) is the author/funder, who has granted bioRxiv a license to display the preprint in perpetuity. It is made available under aCC-BY 4.0 International license.

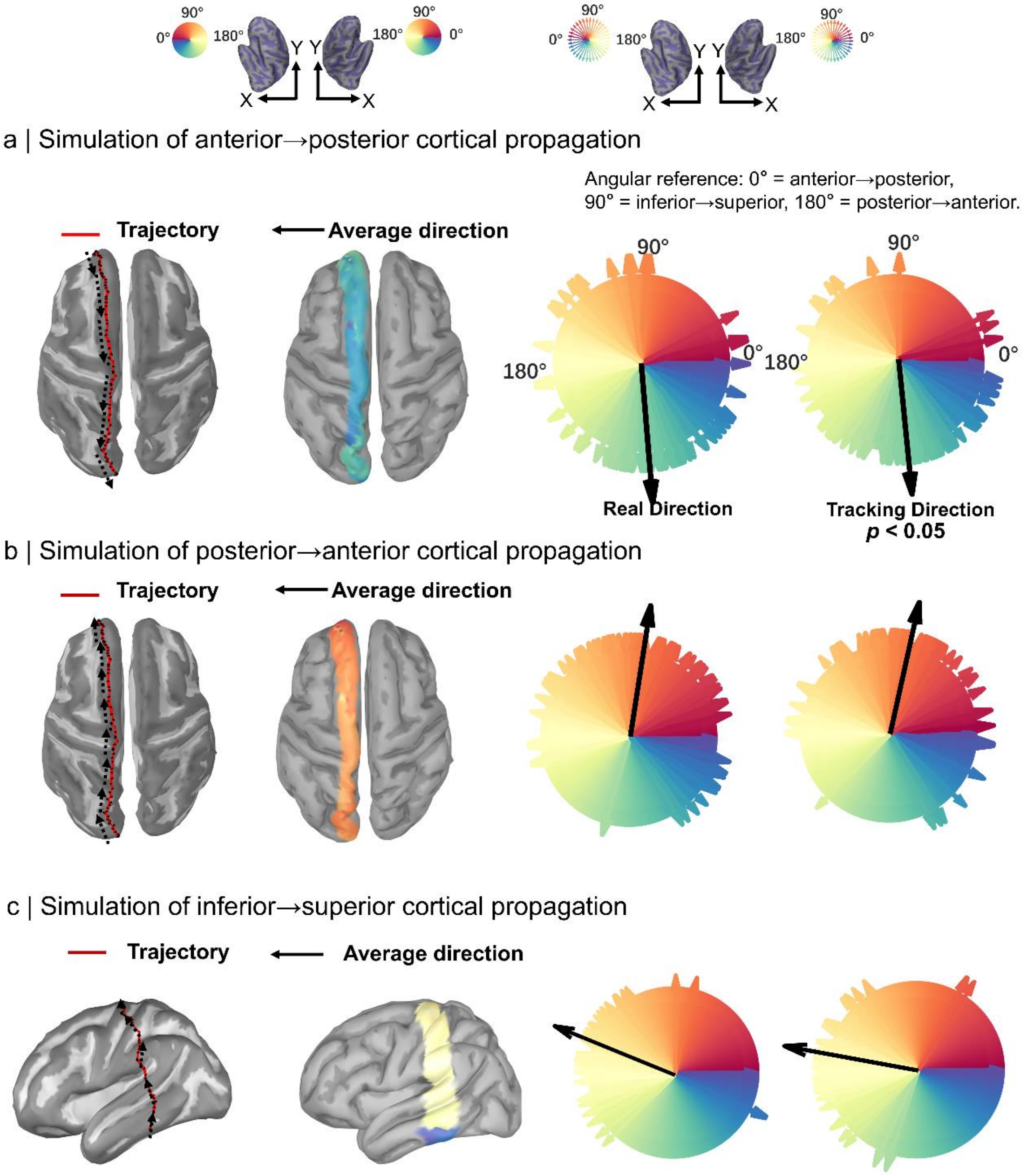

Supplementary Figure 3: Validation of cortical-flow tracking using synthetic propagation patterns.

This figure illustrates validation of the cortical-flow framework using synthetic simulations in which propagation trajectories and directions are known a priori. Synthetic activity patterns were generated by solving the vector heat equation on a Riemannian cortical manifold, producing controlled propagation along predefined anatomical axes. Each simulation therefore provides a ground-truth direction against which estimated propagation can be quantitatively assessed.

a | Anterior→posterior propagation: Activity was initiated in frontal cortex and

bioRxiv preprint doi: https://doi.org/10.64898/2026.03.19.712872; this version posted March 19, 2026. The copyright holder for this preprint (which was not certified by peer review) is the author/funder, who has granted bioRxiv a license to display the preprint in perpetuity. It is made available under aCC-BY 4.0 International license.

propagated toward the occipital pole. The estimated mean propagation angle was 275.69° (95% CI: [259.51°, 291.85°]), with a mean angular error of 3.67°. The rose plot shows strong alignment between tracked and ground-truth directions (Rayleigh test, $p < 0.05$).

b | Posterior→anterior propagation: Activity propagated from occipital to frontal cortices. The estimated mean angle was 86.81° (95% CI: [73.64°, 99.99°]), with a mean angular error of 0.97°, indicating highly accurate directional tracking.

c | Inferior→superior propagation: Activity propagated from ventral temporal toward dorsal parietal cortex, traversing curved, vertically oriented cortical pathways. The estimated mean angle was 157.56° (95% CI: [142.09°, 173.61°]), with a mean angular error of 11.81°, reflecting increased geometric complexity while preserving correct directional alignment.

bioRxiv preprint doi: https://doi.org/10.64898/2026.03.19.712872; this version posted March 19, 2026. The copyright holder for this preprint (which was not certified by peer review) is the author/funder, who has granted bioRxiv a license to display the preprint in perpetuity. It is made available under aCC-BY 4.0 International license.

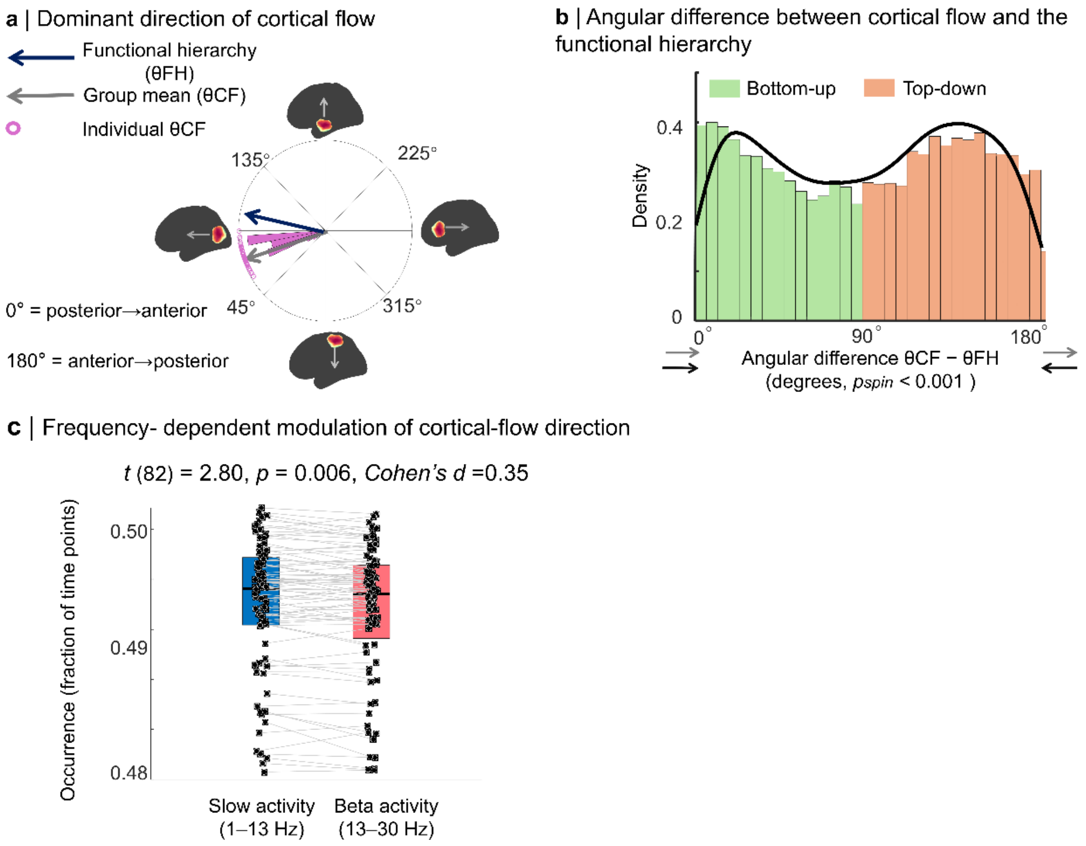

Supplementary Figure 4: Replication of hierarchy-aligned cortical propagation in the OMEGA dataset (N = 83).

a | Dominant direction of cortical flow (θ_{CF}) across OMEGA participants: Polar plot shows individual participant mean cortical-flow directions (gray arrows) relative to the principal functional hierarchy direction (θ_{FH}; blue arrow), revealing a predominant posterior→anterior orientation of spontaneous cortical propagation at the group level.

b | Angular difference between cortical-flow direction and the functional hierarchy: Histogram of $\theta_{CF} - \theta_{FH}$ reveals a robust bimodal distribution ($p_{spin} < 0.001$), with one mode near 0° corresponding to propagation aligned with the functional hierarchy (bottom-up) and a second mode near 180° corresponding to propagation opposed to the hierarchy (top-down).

c | Frequency-dependent modulation of hierarchy-aligned propagation: Paired dot-and-box plots compare the occurrence of hierarchy-aligned propagation for slow activity (1–13 Hz; blue) and beta activity (13–30 Hz; red). Slow activity shows significantly greater hierarchy-aligned propagation than beta activity ($t(82) = 2.80$, $p = 0.006$,

bioRxiv preprint doi: https://doi.org/10.64898/2026.03.19.712872; this version posted March 19, 2026. The copyright holder for this preprint (which was not certified by peer review) is the author/funder, who has granted bioRxiv a license to display the preprint in perpetuity. It is made available under aCC-BY 4.0 International license.

Cohen's d = 0.35), replicating the Cam-CAN result.

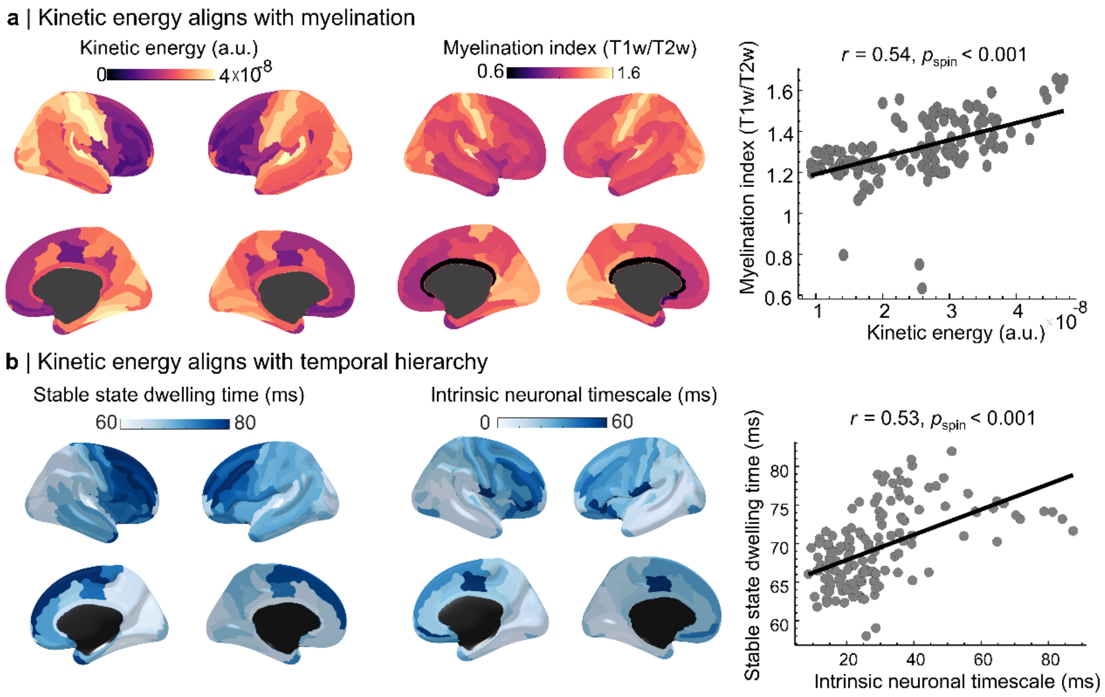

Supplementary Figure 5: Alignment of cortical kinetic energy with myelination and neuronal timescales.

a | Relationship between cortical kinetic energy and myelination: Surface maps show the spatial distribution of kinetic energy and a myelination index derived from T1w/T2w ratios. Vertex-wise analysis reveals a significant positive association between kinetic energy and cortical myelination (r = 0.54, p_{spin} < 0.001), linking propagation strength to underlying microstructural gradients.

b | Relationship between stable-state dwell time and intrinsic neuronal timescales: Surface maps show stable-state dwell time and intrinsic neuronal timescale. Stable-state dwell time correlates positively with regional neuronal timescales (r = 0.53, pspin < 0.001), supporting its alignment with the cortical temporal hierarchy.

bioRxiv preprint doi: https://doi.org/10.64898/2026.03.19.712872; this version posted March 19, 2026. The copyright holder for this preprint (which was not certified by peer review) is the author/funder, who has granted bioRxiv a license to display the preprint in perpetuity. It is made available under aCC-BY 4.0 International license.

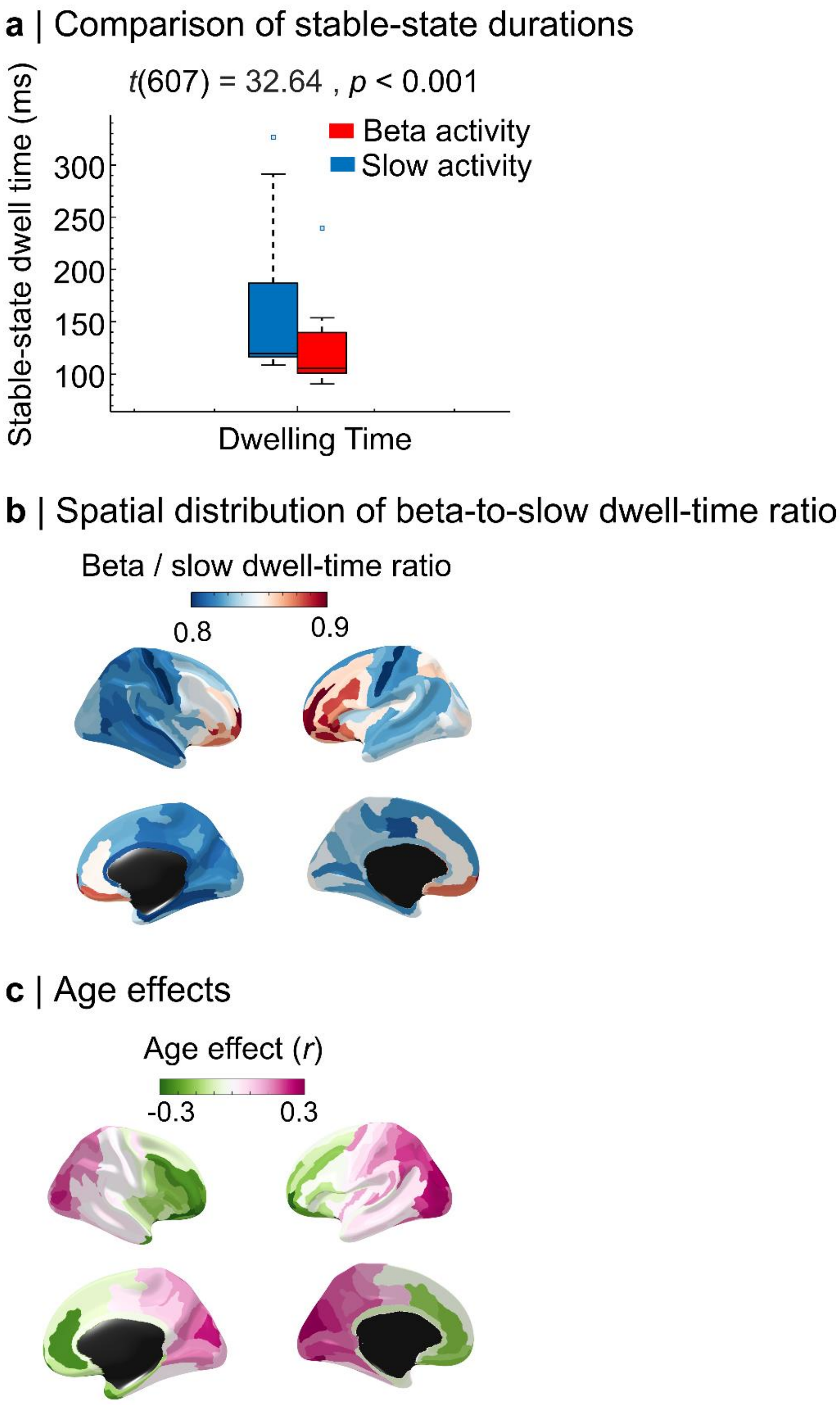

Supplementary Figure 6: Frequency-specific differences in stable-state dwell times and their modulation with aging (Cam-CAN; N = 608).

a | Comparison of stable-state dwell times for slow (1–13 Hz) and beta-band (13–30 Hz) activity: Stable states persist significantly longer for slow activity than for beta

bioRxiv preprint doi: https://doi.org/10.64898/2026.03.19.712872; this version posted March 19, 2026. The copyright holder for this preprint (which was not certified by peer review) is the author/funder, who has granted bioRxiv a license to display the preprint in perpetuity. It is made available under aCC-BY 4.0 International license.

activity ($t(607) = 32.64$, $p < 0.001$), indicating that slower activity supports more sustained cortical states.

b | Spatial distribution of the beta-to-slow dwell-time ratio: Cortical maps show the ratio of beta-band to slow-activity stable-state dwell times across the cortex. Higher ratios in association cortex indicate relatively greater stability of beta-band states compared to slow activity, revealing a spatial organization of frequency-specific stability.

c | Age-related modulation of the beta-to-slow dwell-time ratio: Vertex-wise analysis reveals significant associations between age and the beta-to-slow dwell-time ratio ($p_{FDR} < 0.05$), indicating systematic shifts in the balance between fast and slow stable-state dynamics across the adult lifespan.
